# The phase of plasticity-induced neurochemical changes of high-frequency repetitive transcranial magnetic stimulation are different from visual perceptual learning

**DOI:** 10.1038/s41598-023-32985-8

**Published:** 2023-04-07

**Authors:** Shang-Hua N. Lin, Yun R. Lien, Kazuhisa Shibata, Yuka Sasaki, Takeo Watanabe, Ching-Po Lin, Li-Hung Chang

**Affiliations:** 1 Institute of Neuroscience, National Yang Ming Chiao Tung University, Taipei, Taiwan; 2 Center for Brain Science, RIKEN, Wako, Japan; 3 Department of Cognitive, Linguistics, and Psychological Sciences, Brown University, Providence, USA; 4 Institute of Philosophy of Mind and Cognition, National Yang Ming Chiao Tung University, Taipei, Taiwan

**Keywords:** Visual system, Neuroscience, Inhibition-excitation balance

## Abstract

Numerous studies have found that repetitive transcranial magnetic stimulation (rTMS) modulates plasticity. rTMS has often been used to change neural networks underlying learning, often under the assumption that the mechanism of rTMS-induced plasticity should be highly similar to that associated with learning. The presence of visual perceptual learning (VPL) reveals the plasticity of early visual systems, which is formed through multiple phases. Hence, we tested how high-frequency (HF) rTMS and VPL modulate the effect of visual plasticity by investigating neurometabolic changes in early visual areas. We employed an excitatory-to-inhibitory (E/I) ratio, which refers to glutamate concentration divided by GABA+ concentration, as an index of the degree of plasticity. We compared neurotransmitter concentration changes after applying HF rTMS to the visual cortex with those after training in a visual task, in otherwise identical procedures. Both the time courses of the E/I ratios and neurotransmitter contributions to the E/I ratio significantly differed between HF rTMS and training conditions. The peak E/I ratio occurred 3.5 h after HF rTMS with decreased GABA+, whereas the peak E/I ratio occurred 0.5 h after visual training with increased glutamate. Furthermore, HF rTMS temporally decreased the thresholds for detecting phosphene and perceiving low-contrast stimuli, indicating increased visual plasticity. These results suggest that plasticity in early visual areas induced by HF rTMS is not as involved in the early phase of development of VPL that occurs during and immediately after training.

## Introduction

Visual perceptual learning (VPL) refers to long-term performance enhancement as a result of visual practice and is regarded as a manifestation of visual plasticity. In addition to visual practice, high-frequency (HF) repetitive transcranial magnetic stimulation (rTMS) also induces visual plasticity^1–3^. VPL and the effect of rTMS show similar characteristics. For example, both VPL and the effect of rTMS outlast the period of visual practice and stimulation^4–6^, and may produce a window of long-lasting plastic changes^7–9^. Furthermore, VPL and the effect of rTMS are associated with changes in the visual cortex^9–20^. Interactions between visual training and rTMS have been reported. First, the development of VPL reduces subsequent TMS effects^21^. Second, inhibitory rTMS that follows the encoding of VPL interferes with its consolidation^22,23^. Thus, it is led to the assumption that they may share common neural mechanisms underlying the excitatory modulation of visual training and HF rTMS. However, compared to visual training, the plasticity effect caused by excitatory rTMS can be easily and artificially reversed by subsequent inhibitory rTMS^18^ or can lead to dedifferentiation of preferred neural representations^9^, which contradicts the common belief about the effects of visual training, such as learning specificity. Moreover, several studies have also indicated that VPL may be formed through multiple phases^24–28^, which also raises the question of how the process of HF rTMS is associated with the formation of VPL.

Here, we specifically examined whether neurochemical mechanisms underlying VPL and the effects of HF rTMS are similar. We measured the time course of concentration changes in glutamate (Glu), an excitatory neurotransmitter, and γ-aminobutyric acid (GABA), an inhibitory neurotransmitter, in the human visual cortex using proton magnetic resonance spectroscopy (MRS) after the encoding of VPL and various types of rTMS. We calculated and compared the time course of the E/I ratio (the concentration of Glu divided by the concentration of GABA) as an established index of plasticity^6,8,29,30^ in each of the VPL and TMS condition. Moreover, we observed the response to HF rTMS in cortical excitability and contrast sensitivity in the control experiments to clarify the behavior aftereffects as a comparison to visual training. Aside from the increases in cortical excitability, changes in behavior and the E/I ratio caused by HF rTMS were comparable to those caused by visual training, although the E/I ratio showed inconsistency in terms of the time course and mechanism that responded to increased plasticity that occurs during or immediately after training.

## Results

### E/I ratio changes due to rTMS application and visual training

**Figure 1:**
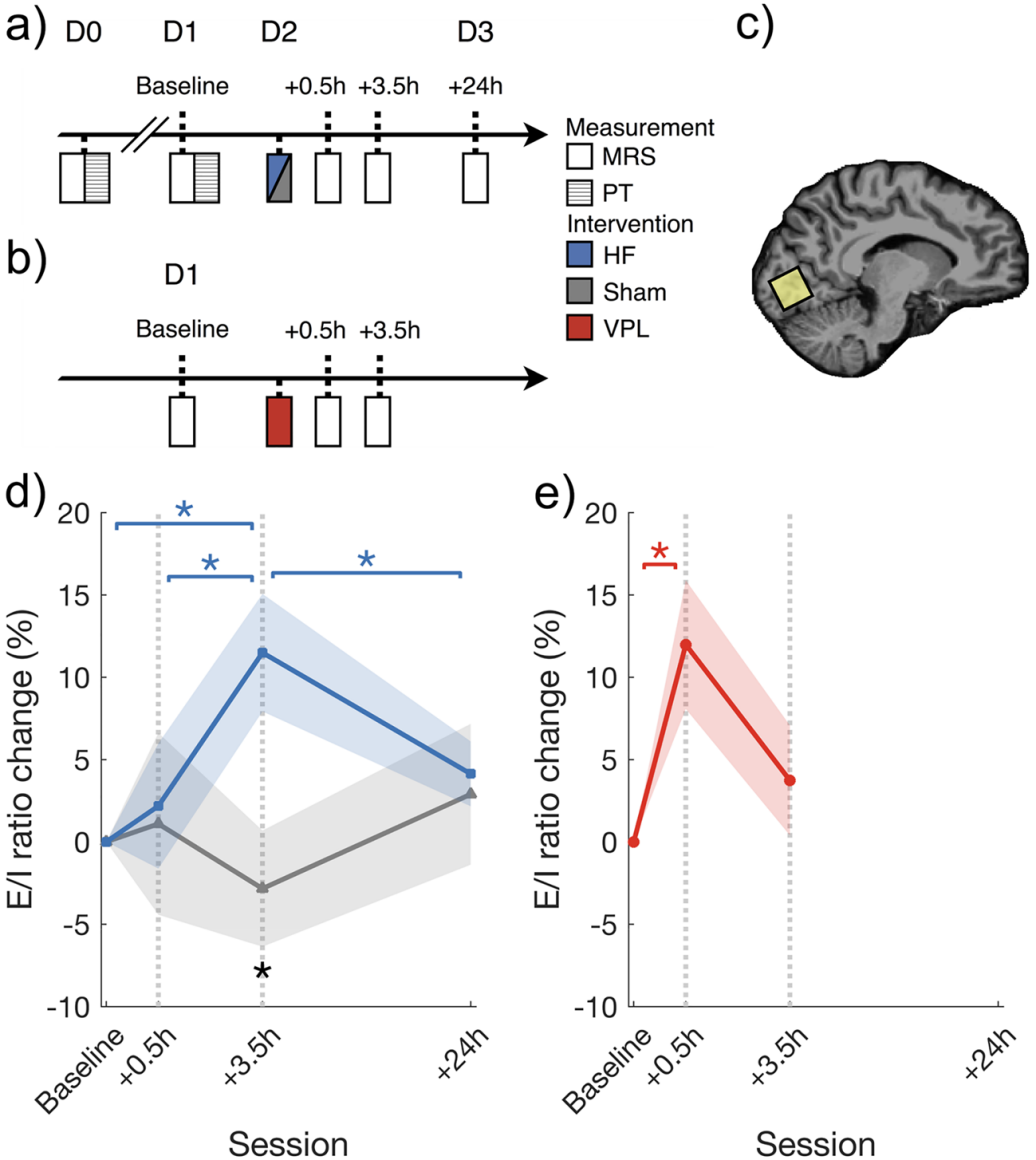
(**a**) Procedures for the HF rTMS groups. (**b**) Procedures for the VPL group^8^. (**c**) Voxel of interest (VOI) in the visual cortex for MRS measurements. (**d**) Mean (± S.E.M.) E/I ratio changes in the HF rTMS (blue) and sham rTMS (gray) groups as a function of time. (**e**) Mean (± S.E.M.) E/I ratio changes in the VPL (red) group as a function of time. The E/I ratio refers to the concentration of glutamate divided by the concentration of GABA+. Shaded areas indicate S.E.M. An asterisk indicates a significance level of *p < 0.05 after FDR correction. The color of the asterisks represents post hoc comparisons: blue asterisks indicate the comparisons within the HF rTMS group, red asterisks indicate the comparisons within the VPL group, and black asterisks indicate the significant differences between the HF rTMS and the other groups. *MRI* magnetic resonance imaging, *MRS* magnetic resonance spectroscopy, *PT* phosphene threshold, *HF* high-frequency, *VPL* visual perceptual learning.

In Experiment 1, we measured the concentrations of Glu and GABA+ and calculated the E/I ratios in the HF rTMS, sham rTMS, and visual training (VPL) conditions. For the HF rTMS group (n = 16) and the sham rTMS group (n = 12), 10-Hz rTMS pulses were applied over the occipital cortex. For the VPL group (n = 12, published data in Ref.^8^), a standard 8-block orientation discrimination task was trained at the center of the visual field. As our previous study showed that the learning of the visual task is consolidated after 3.5 h^8^, MRS sessions were measured at three different time points, including the pre-training/stimulation baseline, as well as 0.5 and 3.5 h after training or stimulation. Furthermore, a fourth time point at 24 h after stimulation was measured to further clarify the overnight aftereffect of rTMS (Fig. 1a,b). The linear mixed-model (LMM) analysis was conducted on E/I ratio changes with the within-subject factor of time (baseline, 0.5 h after, and 3.5 h after stimulation/training, as well as 24 h after stimulation) and the between-subject factor of group (HF rTMS, sham rTMS, and VPL groups). A significant group × time interaction (*F*_5,101.68_ = 2.73, *p* = 0.023, *η*2* p* = 0.12) was found (Fig. 1d,e). No significant effect of group (*F*_2,41.70_ = 1.87, *p* = 0.167, *η*2* p* = 0.08) or time (*F*_3,101.68_ = 1.71, *p* = 0.169, *η*2* p* = 0.05) was observed (for details, see Supplementary Tables S4 and S5). Given the significant interaction between the two factors, we further examined how the E/I ratios changed over time with each of the interventions. At 3.5 h after HF rTMS, the E/I ratio was significantly greater than that at baseline (*t*_15_ = 3.22, *p* = 0.017, after false discovery rate [FDR] correction, Cohen’s *d* = 0.80, 95% CI [7.31 24.45]), and significantly higher than those at 0.5 h (*t*_15_ = 3.22, *p* = 0.017, after FDR correction, Cohen’s *d* = 0.80, 95% CI [5.21, 22.35]) and 24 h (*t*_15_ = 2.60, *p* = 0.041, after FDR correction, Cohen’s *d* = 0.65, 95% CI [4.33, 21.47]). Furthermore, 24 h after stimulation, the E/I ratio change was not significantly different from the baseline value (*t*_15_ = 2.10, *p* = 0.080, after FDR correction, Cohen’s *d* = 0.20, 95% CI [− 5.59, 11.55]). In other words, the E/I ratio reached the highest level after 3.5 h and returned to baseline 24 h after stimulation (Fig. 1d). On the other hand, the E/I ratio change at 0.5 h after training was significantly greater than that at baseline (*t*_11_ = 3.06, *p* = 0.032, after FDR correction, Cohen’s *d* = 0.89, 95% CI [1.48, 22.47]). However, at 3.5 h after training, the E/I ratio change was not significantly different from the baseline value (*t*_11_ = 1.12, *p* = 0.288, after FDR correction, Cohen’s *d* = 0.32, 95% CI [− 6.77, 14.23]). These data indicate that the E/I ratio increased 0.5 h after VPL and rebounded to near baseline 3.5 h after training (Fig. 1e). In contrast to the HF rTMS and VPL groups, no significant differences were found in any comparison within the sham rTMS group (Fig. 1d). Moreover, the additional post hoc analysis showed that at the 3.5 h session, the elevated E/I ratio was significantly higher in the HF rTMS group than in the sham rTMS group (*t*_26_ = 2.86, *p* = 0.0185, after FDR correction, Cohen’s *d* = 1.08, 95% CI [4.01, 24.66]). These results suggested that the time courses of changes in the E/I ratios, which are associated with the degree of plasticity, induced by rTMS and visual training were different.

### Change in each metabolite due to rTMS application and visual training

**Figure 2:**
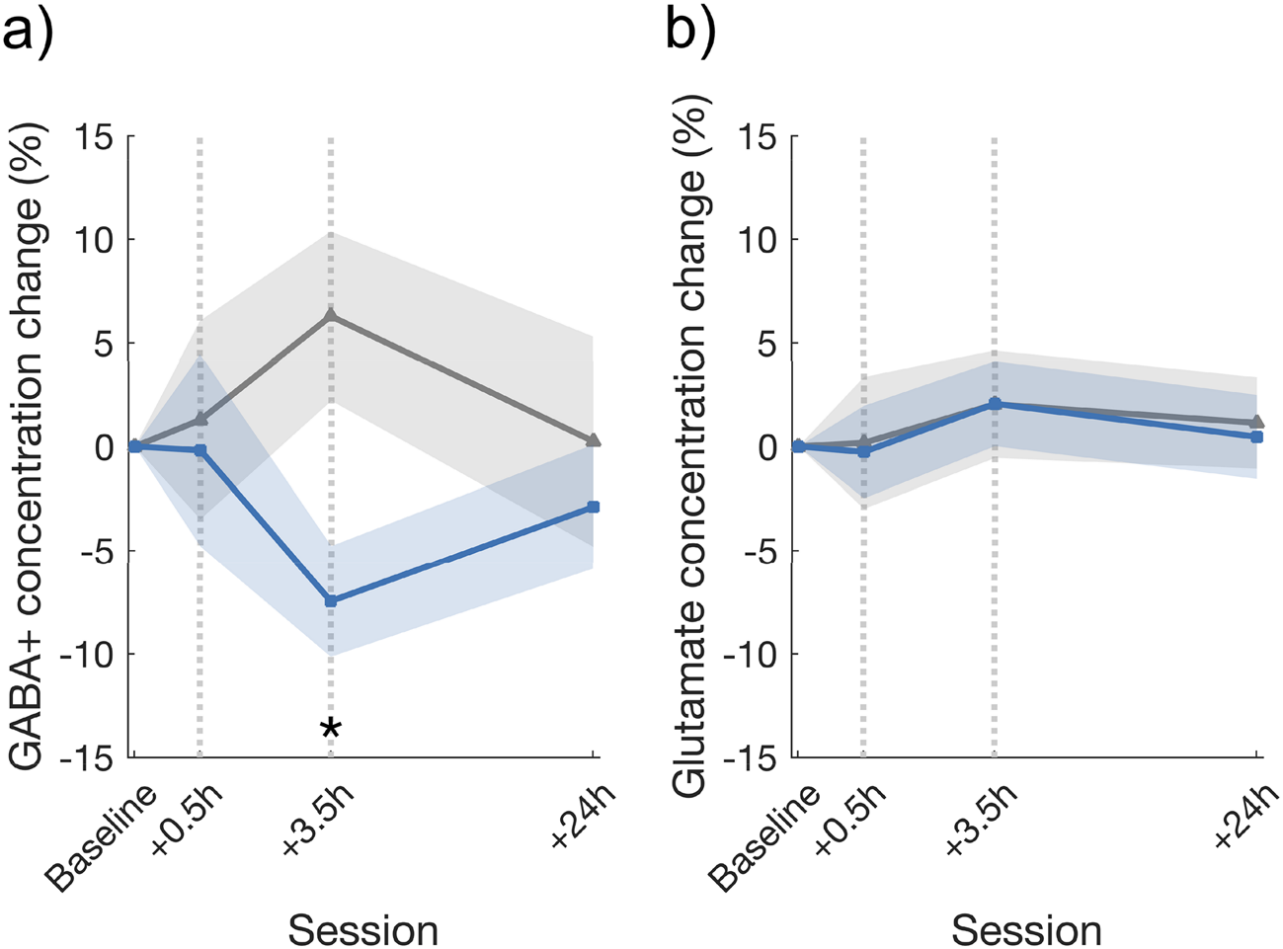
Mean (± S.E.M.) changes in the concentrations of (**a**) GABA+ and (**b**) glutamate across the measured time course in the HF rTMS (blue) and sham rTMS (gray) groups. (**a**) At 3.5 h after stimulation, there was a significant between-group difference in GABA+ level between HF rTMS and sham rTMS groups (*p* = 0.023, FDR corrected). The mean ratio of GABA+ tended to decrease in the HF rTMS group (*p* = 0.014, uncorrected) and increase in the sham rTMS group (p > 0.05, uncorrected) compared to baseline. (**b**) There were no significant differences in the glutamate level. Shaded areas indicate S.E.M. An asterisk indicates a significance level of *p < 0.05 after FDR correction; the color (black) indicates a significant difference between the HF and the other groups in the post hoc comparisons.

To clarify whether the E/I ratio changes from before to after the HF rTMS and VPL interventions were governed by changes in GABA or glutamate, further analysis of the individual metabolites was conducted using LMM with the within-subject factor of time (baseline, 0.5 h after, and 3.5 h after stimulation/training, as well as 24 h after stimulation) and the between-subject factor of group (HF rTMS, sham rTMS, and VPL groups). Regarding the GABA+ levels, the results showed a non-significant interaction between group and time (*F*_5,101.35_ = 2.11, *p* = 0.070, *η*2* p* = 0.09) and no significant change based on group (*F*_2,40.77_ = 1.48, *p* = 0.239, *η*2* p* = 0.05) or time (*F*_3,103.73_ = 0.30, *p* = 0.825, *η*2* p* < 0.01). As the elevated E/I ratios were different among groups, especially at the 3.5 h session, there was a significant fixed effect on GABA+ when compared the other groups to the sham rTMS group at the 3.5 h session (*t*_101.35_ = 2.39, *p* = 0.018) (for details, see Supplementary Tables S6 and S7). Indeed, the between-group comparison also showed that at the 3.5 h session, the decreased GABA+ level was significantly lower in the HF rTMS group than in the sham rTMS group (*t*_26_ = − 2.82, *p* = 0.023, Cohen’s *d* = − 1.10, 95% CI [− 23.94, − 3.56], after FDR correction) (Fig. 2a). Regarding glutamate levels, the results showed neither a main effect of time (*F*_3,100.92_ = 1.70, *p* = 0.172, *η*2* p* = 0.05), a main effect of group (*F*_2,39.74_ = 1.22, *p* = 0.306, *η*2* p* = 0.06) nor an interaction between the two factors (*F*_5,100.92_ = 0.67, *p* = 0.648, *η*2* p* = 0.03) was observed (for details, see Supplementary Tables S8 and S9) (Fig. 2b). Although the GABA+ change was obvious in the HF rTMS group, no change was observed in the glutamate level. In contrast, a significant increase in the glutamate level was observed only in the VPL group (3.5 h vs. baseline: *t*_11_ = 2.84, *p* = 0.048, Cohen’s *d* = 0.82, 95% CI [1.16, 9.14], after FDR correction) (Supplementary Fig. S4). These results indicate that changes in major metabolites induced by HF rTMS and visual training were significantly different. In summary, the significant differences both in the peak time of changes in E/I ratios over the examined time course and in the major metabolites that were changed after HF rTMS and visual training suggested that the underlying mechanisms of the plasticity caused by HF rTMS and by visual training were not the same.

### Changes in cortical excitability and perceptual sensitivity by rTMS

**Figure 3:**
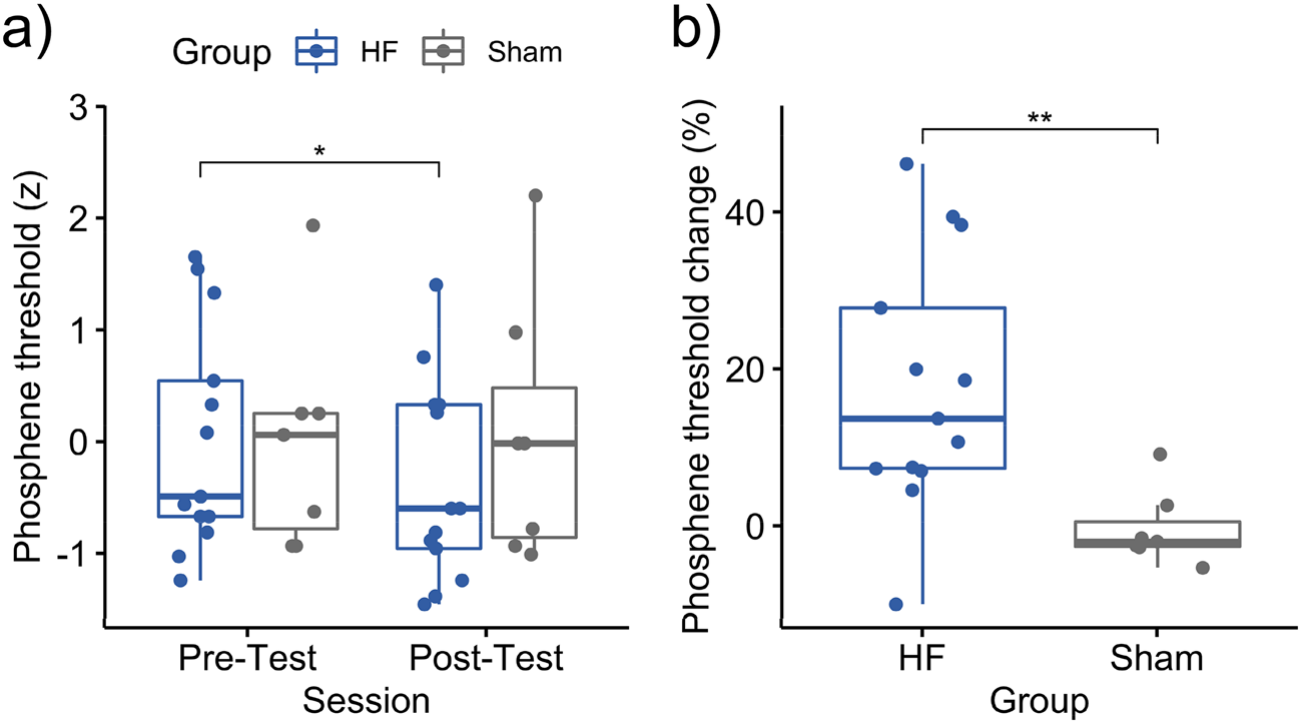
Phosphene threshold (PT) changes after HF rTMS and sham rTMS. A greater percentage of PT changes refers to a lower threshold at the post-test when compared to the individual baseline. A lower threshold indicates that a lower intensity of TMS is needed to elicit phosphenes. The HF rTMS group shows significantly greater change in PT than the sham rTMS group. Each dot represents an individual participant. Asterisks indicate the significance levels: *p < 0.05, **p < 0.01.

**Figure 4:**
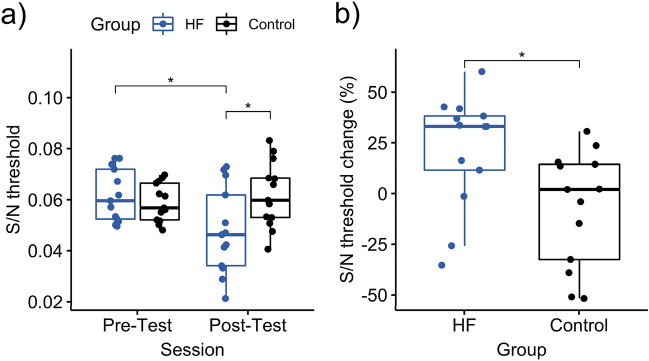
The orientation discrimination thresholds before and after intervention (HF rTMS group) and compared to the rest (control group). A lower S/N threshold suggests better performance, indicating that the participant could detect Gabor patch at a lower stimulus contrast. Each dot represents an individual participant. An asterisk indicates the significance level of *p < 0.05 after FDR correction. *S/N* signal-to-noise.

To test whether the HF rTMS influences on the visual task, two control experiments were additionally conducted. The Experiment 2 tested the phosphene threshold (PT) to single pulse TMS as a measure of visual cortical excitability. The Experiment 3 tested whether the rTMS modulates the sensitivity to orientation perception by obtaining the signal-to-noise threshold (S/N threshold). The PT was significantly decreased in the HF rTMS group (*t*_12_ = 2.84, *p* = 0.015, Cohen’s *d* = 0.39, 95% CI [0.09, 0.66]) while the threshold in the sham rTMS group remained largely unchanged (*t*_6_ = − 0.47, *p* = 0.654, Cohen’s *d* = − 0.06, 95% CI [− 0.37, 0.25]) (Fig. 3a). The change of PT also showed a congruent result (*t*_15.4_ = 3.25, *p* = 0.002, Cohen’s *d* = 1.34, 95% CI [7.82, 28.42]) (Fig. 3b). The S/N threshold decreased significantly in the HF rTMS group (*t*_12_ = 3.05, *p* = 0.01, Cohen’s *d* = 0.98, 95% CI [0.01, 0.02]) but no such finding occurred in the sham rTMS group (*t*_12_ = − 0.67, *p* = 0.519, Cohen’s *d* = − 0.28, 95% CI [− 0.01, 0.01]) (Fig. 4a). The change of S/N threshold also showed a congruent result (*t*_24.0_ = 2.62, *p* = 0.015, Cohen’s *d* = 1.28, 95% CI [6.15, 51.70]) (Fig. 4b). These results confirm the general thought assumption that HF rTMS can produce excitatory plastic aftereffects.

## Discussion

In the present study, we demonstrated significant differences in neurochemical changes in the human visual cortex associated with HF rTMS and visual training. Both HF rTMS and visual training increased visual plasticity, as we assumed they would. However, we also found significant differences in the time course of the plasticity changes and in the major metabolite involved in the increased plasticity associated with HF rTMS effects and VPL. These results suggest that the underlying neurochemical processes associated with HF rTMS in early visual areas does not reflect an early phase of plasticity that occurs during and immediately after training. With regard to the time course of the E/I ratio changes, we found that the E/I ratio peaked at 0.5 h and returned to baseline at 3.5 h in the VPL group. In contrast, the E/I ratio did not significantly increase at 0.5 h but peaked at 3.5 h after the HF rTMS stimulation. With regard to the metabolites that played a major role in the E/I ratio changes, we found that for the HF rTMS group, the increases in the E/I ratio were mostly attributable to reductions in GABA+, whereas for the VPL group, the increases in the E/I ratio largely depended on increases in glutamate.

Our results showed that the changes in the E/I ratio occurred much later in HF rTMS stimulation than in visual training. Previous research has demonstrated that HF rTMS induced a robust and long-lasting strengthening of structural and functional changes of excitatory postsynapses at 2–4 h post-stimulation in mouse entorhinohippocampal slice cultures^31–33^. Similar results were obtained when using voltage-sensitive dyes, which visually evoked responses lasting for 2–3 h after HF rTMS and were linked to the weakened inhibition function counteracting the excitatory driving input^18^. Moreover, HF rTMS caused a long-lasting change in orientation representation (lasted for more than 60 min) where visual cortical maps were unstable and responsive to input biases^9^, and increased immediate early gene expression proteins c-Fos and zif268 (after 2 h), both of which are important molecular markers of synaptic plasticity^34,35^. In congruence with those brain stimulation studies, our results demonstrated that the HF rTMS-induced E/I ratio changes seems to reveal a long-term excitatory bias rather than a short-term change induced by visual learning^29^.

Thus, our results suggest that at least rTMS applied to early visual areas induces plasticity that is not exactly the same as the plasticity during and immediately after VPL training. Then, why did the changes in the neurochemical environment induced by HF rTMS occur much more slowly than the changes induced by visual perceptual learning?

A number of studies of VPL have pointed out that VPL is developed through multiple phases^28,36^. For example, according to Sagi and Karni (1993), during and immediately after training, intraocular transfer of learning the results is complete and no location specificity is observed^24^. However, hours later, intraocular transfer became incomplete suggesting that the learning proceeded from higher stages to the lower cortical stages perhaps involving early visual areas and location specificity. Moreover, incomplete intraocular transfer and location specificity remained for more than one year. These results have suggested that VPL is developed through at least two phases, early and later phases: The early phase occurs during or immediately after training and is followed by the later phase that occurs a few hours later. The later phase is involved in maintaining the learning on a long-term basis. The E/I ratio elevation we observed matches the time course of the later phase of the development of VPL. Accordingly, we inferred that the plasticity in the early visual cortex caused by HF rTMS does not correspond to the early phase of plasticity induced by VPL, but rather to its later phase, which is more related to the long-standing retention of VPL.

Another major difference is that the VPL groups had exposed to visual stimulus whereas the HF rTMS group did not. The E/I ratio changes induced by the learning-dependent manner in the VPL group may facilitate and gate the plasticity temporal window with the top-down regulation boosted by attention in the visual system^37^, although some evidence also showed that VPL was associated with local axonal sprouting and pruning^38^ and long-lasting potentiation of synaptic transmission^14^ in the primary visual cortex. Despite debate over the mechanisms of VPL, the plastic effects after learning have been widely reported. The effect of visual training is susceptible to interference during the course of two consecutive tasks with similar features^7^, and it appears to be associated with the consolidation stage of that learning^39,40^. On the other hand, unlike visual training, the HF rTMS here was applied without any external visual signal input, the plastic effect occurs mainly as a result of neural oscillation and synaptic changes. It may explain why rTMS-induced E/I regulation might remain in a more plastic and unstable state reflected by a slower E/I regulation process than the VPL group. We suggest that systematic research is necessary to discuss this issue.

In addition, not only the timing of the E/I ratio changes, but also the major metabolite contributing the change of E/I ratio differ between the HF rTMS and VPL, while both the HF rTMS and VPL groups showed the elevation in the E/I ratio. Our results demonstrated that the increases in the E/I ratio were attributed to the reduction of GABA+ for the HF rTMS group but to the increase of glutamate for the VPL group. In the HF rTMS group, we found that HF rTMS-caused changes in GABA+ concentration were more prominent than changes in glutamate, and the dynamic changes in the E/I ratio were mostly driven by inhibitory neurotransmission, GABA, which may be mediated by intracortical inhibition^18^ or the reduction of GABAergic interneuron synapses^33^ in early visual areas. The source of MRS-GABA+ levels is likely related to either the extra-synaptic GABAergic tone^41,42^ or the intracellular GABA^43^, although recent studies have found inconsistent results^44,45^. In addition, similar results on the reduction of GABA+ were found using different noninvasive stimulation protocols in the motor cortex, such as continuous theta burst stimulation (TBS)^46^, anodal transcranial direct current stimulation (tDCS)^47–49^, and cathodal tDCS^50^. Although we did not find any differences in glutamate levels when compared to the sham rTMS group, evidence has shown that HF rTMS induces alterations that are consistent with the LTD of inhibitory neurotransmission^33^ and LTP of excitatory neurotransmission in the visual cortex^51^, motor^52,53^, and hippocampus^31,32^. On the other hand, our results in the VPL group demonstrated that the increases in the E/I ratio were mainly attributed to the increases in glutamate during the visual training. One possible explanation is that the repetitive visual presentations during visual perceptual learning increase the fluctuations in excitatory neurotransmitter levels or increase the glutamatergic excitatory synaptic strength in neurons. Previous studies have shown that prolonged visual exposures elevate the levels of glutamate, suggesting that glutamate levels increase with the amount of visual processing in the visual system^54–56^. Moreover, the associated cortical changes after learning have also been linked with synaptic plasticity modulation and shown glutamatergic dependency^57–59^. On the contrary, alteration in GABA level has, however, been linked to enhanced perception and an unstable state as well^60^. Based on the current evidence, both forms of neurochemical change can contribute to visual plasticity. In addition, since the level of GABA+ and glutamate may also change depending on when it is measured. For instance, the elevated GABA+ was accompanied by elevated glutamate or vice versa in the control groups, resulting in relatively stable E/I ratios across time. Meanwhile, it should be noted that the metabolic pathways regulating the synthesis and cycling of GABA and glutamate/glutamine have been tightly coupled, and glutamine generated through this cycle is the primary precursor for the synthesis of GABA^61^. Accordingly, considering both GABA and glutamate concentration could better address the dynamic balance of excitation and inhibition neurotransmission for plasticity and stability in the brain^30^.

Although our results demonstrated that HF rTMS-induced changes in the E/I ratios reflected plasticity, one may wonder whether the E/I ratio change reflects the brain plasticity. First, the sham rTMS group did not show any significant E/I ratio changes after the stimulation as the control of the effect on artificially induced neuromodulation. On the other hand, we also observed that the phosphene threshold and the contrast sensitivity were temporally improved in the HF rTMS group when compared to the control group. In addition, the patterned rTMS protocols are the most commonly used paradigms to induce plasticity in the human cortex^62–64^. Among them, the 10 Hz HF rTMS is one of the standard protocols and is widely applied in the visual cortex. One may wonder why 10 Hz rTMS can selectively enhance the plasticity in the visual cortex. Previous studies have demonstrated that rTMS-induced excitability and spontaneous oscillations in the visual cortex are produced by the same neuronal mechanisms^65^. Research has shown that 10 Hz selectively increases visual evoked potential^19^, and the visual phosphene perception induced by 10 Hz rTMS is associated with the same power^66^, phase^67,68^, and prestimulus oscillatory neural activity in the alpha band (~ 10 Hz). The effect of 10 Hz rTMS in the visual areas replicated existing studies despite the fact that the parameters may not be identical. On the other hand, HF rTMS transiently enhanced contrast sensitivity as well and such improvement was comparable to that achieved by standard visual training^8^.

In summary, we investigated whether the aftereffect of HF rTMS and VPL share common underlying neurochemical processes. Our results indicated that although both HF rTMS and visual training enhanced visual plasticity as evidenced by E/I changes in early visual areas, the time courses and the major metabolites involved in the increased plasticity for HF rTMS effects and VPL were dissociated. The finding of differences in the timing of plasticity increases and neurochemical mechanisms may offer more insight into future applications of rTMS and visual training.

## Methods

### Participants

A total of 102 healthy volunteers were recruited and randomly assigned to the following groups. Twenty-eight participants were included in the analysis in Experiment 1 in either the high frequency (HF, n = 16, age = 23.4 ± 2.66, 9 females) or sham (n = 12, age = 25.7 ± 5.09, 9 females) stimulation groups. For comparison, twelve healthy volunteers were included in the VPL group (adapted from Ref.^8^). Twenty participants were included in Experiment 2 in the HF rTMS (n = 13, age = 23.4 ± 2.22, 7 females) or sham rTMS (n = 7, age = 23.9 ± 4.06, 2 females) groups. Twenty-six participants were included in Experiment 3 in the HF rTMS (n = 13, age = 23.7 ± 2.95, 11 females) or control (n = 13, age = 24.4 ± 3.20, 10 females) groups. Participants reported no history of neurological, medical, visual, or memory disorders. Twenty-eight subjects were excluded due to the absence of phosphene perception (drop out at Day 1, n = 8), poor data quality (drop out at analysis stage, n = 12), or failure to consistently produce phosphenes (n = 8). None of the following factors showed a difference between groups: age, sex, dominant hand/eye, education, processing speed, divided attention, or selective attention. The project was approved by the institutional review board of National Yang Ming Chiao Tung University and Brown University. All participants gave written informed consent. All experiments were performed in accordance with relevant guidelines and regulations.

### Experimental procedures

In Experiments 1, 2, and 3, a session of rTMS intervention was applied during the experiment. In Experiment 1 (MRS experiment), four MRS scans (baseline, 0.5 h, 3.5 h, and 24 h after stimulation) were conducted in the rTMS groups; furthermore, three MRS scans (baseline, 0.5 h, and 3.5 h after training) were acquired from the VPL group to investigate the neurochemical mechanisms of induced plasticity after rTMS or visual training. In addition the neurochemical measurements, two behavior experiments were also performed to confirm the modulatory aftereffects of rTMS. In Experiment 2, we investigated the change in cortical excitability after rTMS using the phosphene threshold. Experiment 2 consisted of two test sessions in one day: pre-test, stimulation, and post-test. In Experiment 3, we measured the change in visual performance after rTMS by the contrast thresholds for detection of the Gabor patch, which was also used for visual training. The procedures were identical to those of Experiment 2.

### Phosphene thresholds

TMS was performed using a 70-mm air-cooled figure-eight coil with a Magstim Rapid^2^ stimulator (The Magstim Company Ltd., Whitland, UK) as well as the D70 Alpha Flat Coil for sham intervention. Stimulation sites for each participant were defined in the upper quadrant of the visual field and measured by retinotopic mapping analysis^69^ using FreeSurfer. The coil was fixed over the predefined target marked on the scalp. The coil was placed tangentially to the skull, parallel to the O1-Oz-O2 (10–20 international system) line with the handle pointing outwards. The participants were adapted to the dark environment and instructed to not expect visual change, to keep their eyes open, to maintain visual fixation on the central spot and, after each TMS pulse, to report the presence or absence of a phosphene. The phosphene threshold (PT) was defined by the minimal stimulation output intensity to induce a 50% chance of reporting phosphenes. The participants were familiarized with the TMS procedure and phosphene perception and ran at least one threshold estimation before the beginning of the experiment.

### Repetitive transcranial magnetic stimulation

In the rTMS groups (Experiments 1, 2, and 3), the participants were assigned to either the active suprathreshold high-frequency (10-Hz rTMS with the stimulation intensity representing 100–120% of the average PT in pretests) or inactive sham (the same protocol, but using a sham coil instead) rTMS groups. rTMS protocols consisted of 30 trains of pulses (10 Hz for 2 s), with an intertrain interval of 10 s for a total of 600 pulses.

### Visual perceptual task and training

In the VPL group (Experiment 1) and control group (Experiment 3), participants performed the two-interval forced-choice orientation discrimination task (see Ref.^8^). In each trial, two intervals, either a Gabor patch with a certain S/N ratio or a noise pattern (0% S/N ratio), were presented in a random sequence. The participants were asked to respond in which interval (first or second) the Gabor patch appeared by pressing buttons. No feedback was provided on their responses. The signal-to-noise threshold (S/N threshold) for each block was measured using the two-down-one-up staircase rule, leading to converging around the 70.7% accuracy rate. In the VPL group, the participants performed the orientation discrimination task with one orientation (trained orientation) for 8 blocks. The trained orientation was selected from among three orientations (10°, 70° or 130°, each ± 60° apart) and counterbalanced across participants. The remaining two orientations served as untrained orientations.

### Behavior data analysis

The phosphene threshold and S/N threshold were used to measure behavior performance in Experiments 2 and 3, respectively. The threshold refers to the level of stimulation strength at which the participant perceives the phosphene or Gabor patch—that is, is more sensitive to the stimulation when the threshold is lower. We measured the percentage change in the thresholds using the following formula.

$$Percentage \; change=\frac{(PreTest-PostTest)}{PreTest}\times 100\%.$$

### ^1^H MRS

In Experiment 1, four MRS sessions were conducted in the rTMS groups: 1 day before (Day 1), 0.5 h after (Day 2), 3.5 h after (Day 2), and 24 h after (Day 3) rTMS intervention. Meanwhile, three MRS sessions were conducted in the VPL group: pretraining baseline (Day 1), 0.5 h after (Day 1), and 3.5 h after (Day 1) training. The rTMS groups were conducted on a 3 T MR scanner (Siemens) with a 32-channel head matrix coil at National Yang Ming Chiao Tung University. The VPL group was conducted on a 3 T MR scanner (Siemens) with a 32-channel head matrix coil in the Brown University MRI Research Facility. The participants were brought to the scanning room immediately after stimulation or training. The procedure for positioning, structural scanning, voxel placement, and shimming took approximately 20–30 min; therefore, the MRS sessions following stimulation or training are referred to as sessions at 0.5 h (no break) and 3.5 h (2-h break following the previous MRS session). Each session took approximately 1 h. High-resolution (1 × 1 × 1 mm^3^) magnetization-prepared rapid gradient echo (MP-RAGE) T1-weighted anatomical brain structure images were acquired (TR/TE/TI = 3500/3.5/1100 ms; FOV = 256 × 256 × 192 mm^3^) to facilitate accurate MRS voxel positioning and for post hoc within-MRS voxel tissue-type segmentation. Based on the individual high-resolution anatomical brain structure, the visual region of interest (ROI) (2 × 2 × 2 cm^3^) was manually placed on the posterior part of the occipital lobe (Fig. 1c) to ensure that the ROI would cover the unilateral primary visual areas in the rTMS groups (Experiment 1) (only the hemisphere of the predefined target was included) and bilaterally (centered at the midline) in the VPL group. This ROI position was carefully replicated during the following MRS sessions by referring to the picture that showed both the anatomical brain structure and the ROI position at baseline. The ROIs overlapped by more than 99% in volume across the MRS sessions in the present study. Shimming was automatically performed using the Siemens advanced shimming algorithm, and further manual adjustments were made to achieve better homogeneity of the magnetic field. The mean (± s.e.m.) shim value (water linewidth) across participants was 14.60 ± 1.02 Hz. The GABA scans were conducted using a GABA-edited MEGA-PRESS sequence (TR/TE = 1500/68 ms, with the editing pulses applied at 1.9 ppm during the edit-on acquisition and 7.4 ppm during the edit-off acquisition, 256 pairs of averages, yielding a total acquisition time of 12 m 54 s), which was used to simultaneously suppress the creatine signal and edit the γ-CH2 resonance of GABA at 3 ppm. The glutamate scans were conducted using the PRESS sequence (TR/TE = 3000/30 ms, 128 averages, yielding a total acquisition time of 6 m 24 s).

### MRS analysis

The GABA levels were obtained from the “difference” spectra of the MEGA-PRESS sequence, all other reported metabolite levels came from the PRESS sequence. We applied the LCModel (http://www.lcmodel.com/lcmodel.shtml) algorithm to all single-voxel ^1^H-MRS data. The spectral range for analysis was set to 0.2–4.2 ppm for the PRESS spectrum and 1.95–4.0 ppm for MEGA-PRESS spectrum. GABA measurements from the MEGA-PRESS spectra included contributions from co-edited resonances from macromolecule resonances at 3.0 ppm and, hence, are represented as GABA+. The control parameter sptype = “mega-press-3” was used for the MEGA-PRESS spectrum. Signal-to-noise ratios (SNR) and linewidths from LCModel were used to enssure data quality. Only those with a full width at half maximum (FWHM) of ≤ 0.1 ppm and an SNR of ≥ 30 for the PRESS scans and an SNR of ≥ 10 for the MEGA-PRESS scans were included for further analysis. The resulting average water FWHM (mean ± s.e.m.) was 0.060 ± 0.016 ppm for MEGA-PRESS scans and 0.043 ± 0.007 ppm for PRESS scans across participants. For both GABA+ and glutamate, the basic functions provided by the LCModel modeled all of the multiplets produced by each metabolite (Supplementary Fig. S1). Note that glutamate and glutamine were separately fitted by the LCModel and that the concentration of glutamate was used for the calculation of E/I ratio changes. The Cramér–Rao lower bounds (CRLB or % s.d.) were used as a measure of quantification of GABA+ and glutamate. Only metabolites with CRLB ≤ 10% were included. The mean (± s.e.m.) CRLB percentage was 6.26 ± 1.02% for GABA scans and 5.34 ± 1.08% for glutamate scans across participants (for details, see Supplementary Fig. S2 and Supplementary Table S1). Because NAA was quite stable (Supplementary Fig. S3), we expressed GABA+ and glutamate metabolite concentrations as a ratio to the reference NAA (*N*-acetylaspartate) metabolite concentration in each MRS session and referred to them as the concentrations of GABA+ and glutamate. Structural images acquired in the same session, as the ^1^H MRS data were tissue segmented to obtain measures of the within-voxel gray matter (GM), white matter (WM) and cerebrospinal fluid (CSF) content for each participant. The tissue components were also to verify the location of VOI across sessions. An E/I ratio change, E/I_change_, during each of the MRS sessions was calculated for each subject according to a formula following the approach of a previous study^8,29^.

$$\frac{E}{I}_{change}\left(t\right)= \left(\frac{\frac{Glu\left(t\right)}{GABA\left(t\right)}}{\frac{Glu\left(1\right)}{GABA\left(1\right)}}-1\right) \times 100\%.$$

Here, GABA + (*t*) and Glu(*t*) represent the concentrations of GABA+ and glutamate, respectively, at a certain MRS session *t* (1 = baseline, 2 = 0.5 h, 3 = 3.5 h, 4 = 24 h); thus, an E/I ratio change of 0% is reported for the baseline session (for details, see Supplementary Table S2).

### Statistical analyses

All of the statistical analyses were conducted using R (version 3.6.2)^70^. We used a linear mixed-effects models (LMM) analysis^71^, taking into account the within-subject and within-group differences. LMM works with unbalanced repeated measures data sets. LMM were fit using the lme4 package^72^ with p values estimated using the lmerTest package^73^. The fixed effects were the group, the time, and the interaction between group and time (Supplementary Table S3). The random effects were the intercepts for the subjects. Models were fitted using restricted maximum likelihood (REML) and *p*-values were obtained using Type III analysis of variance with the Satterthwaite approximations for degrees of freedom, leading to acceptable Type 1 error rates^74^. Significant fixed effects or interactions were followed up with comparisons of estimated marginal means using the emmeans package^75^. Post hoc pairwise comparisons were calculated using *t*-tests with pooled standard deviation. A two-tailed *p*-value < 0.05 was considered to be statistically significant. When a correction for multiple comparisons was needed, the FDR correction was applied. When a test indicated statistical significance, the effect size is shown using *η*2* p* for LMM and Cohen’s *d* for t-tests. Effect sizes of 0.2, 0.5, and 0.8 are termed small, medium, and large, respectively.

## Supplementary Information

Supplementary Information.

## Data Availability

Data file (Data set name: SourceData.xlsx) are available at https://osf.io/download/zk9an/?view_only=8337750116664c16a5be6365747c9aa8.

## Code availability

The computer codes are available from the corresponding author upon request.
