# Supplementary material for: The phase of plasticity-induced neurochemical changes of high-frequency repetitive transcranial magnetic stimulation are different from visual perceptual learning
Source: Sci Rep. 2023 Apr 7;13:5720. doi: 10.1038/s41598-023-32985-8 (PMC10082079; doi:10.1038/s41598-023-32985-8)
Supplement: Supplementary file 1 — Supplementary Information. [file 41598_2023_32985_MOESM1_ESM.docx]

**
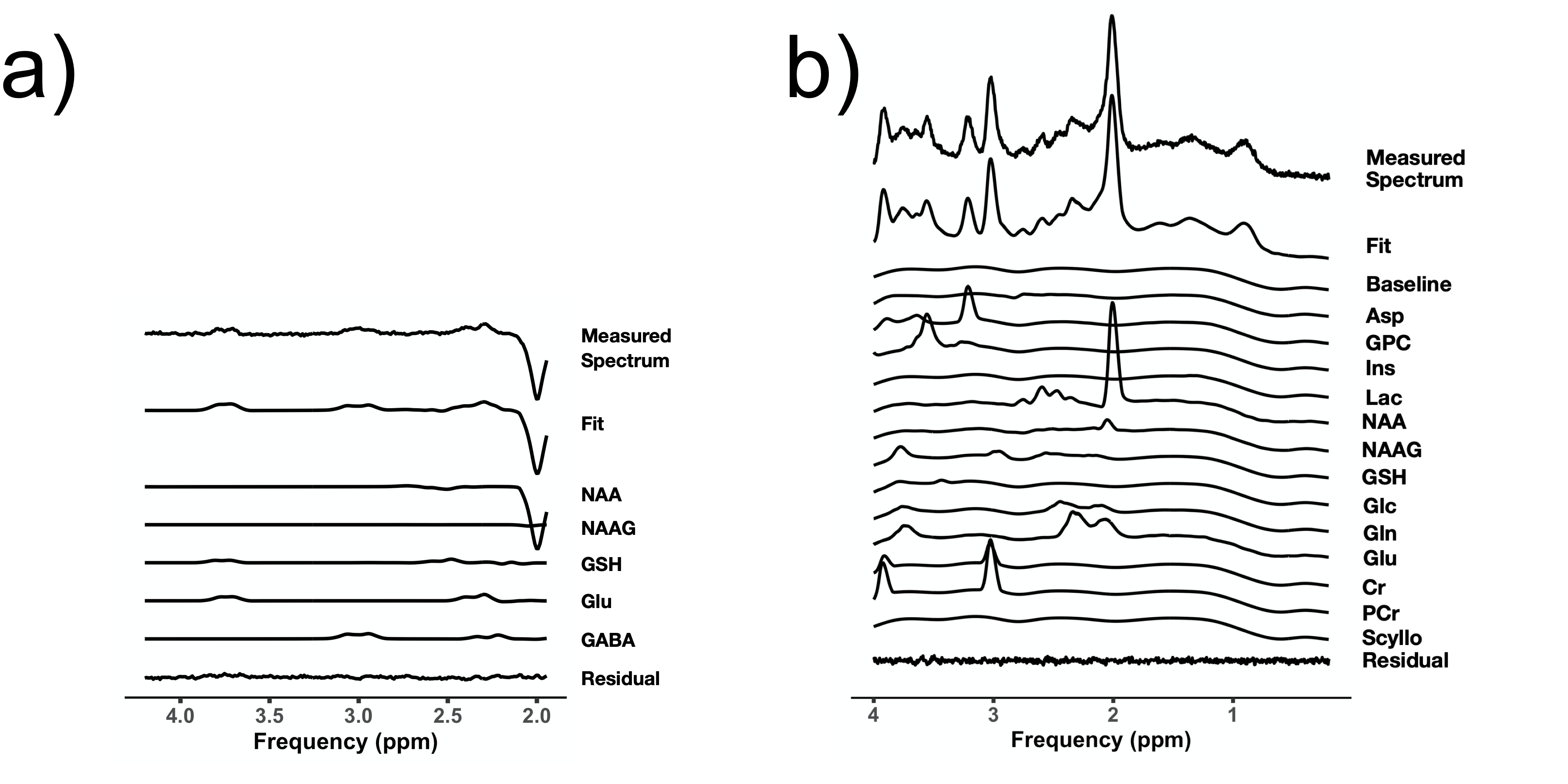
**

**Supplementary Figure 1.** Example of ^1^H-MRS spectra at the early visual area.

a) An example spectrum from the GABA+ (MEGA-PRESS) scan. b) An example spectrum from the glutamate (PRESS) scan. The measured spectra are shown in the first row. The second row indicates the spectra fitted with the LCModel. The bottom row shows the residual remaining after the fitting. The remaining rows show the individual fits for all metabolites from the given acquisition.

NAA, N-acetylaspartate; NAAG, N-acetylaspartylglutamate; GSH, glutathione; Glu, glutamate; GABA, gamma-aminobutyric acid; Asp, aspartate; GPC, glycerophosphocholine; Ins, myo-inositol; Lac, lactate; Glc, glucose; Gln, glutamine, Cr, creatine; PCr, phosphocreatine; Scyllo, scyllo-inositol


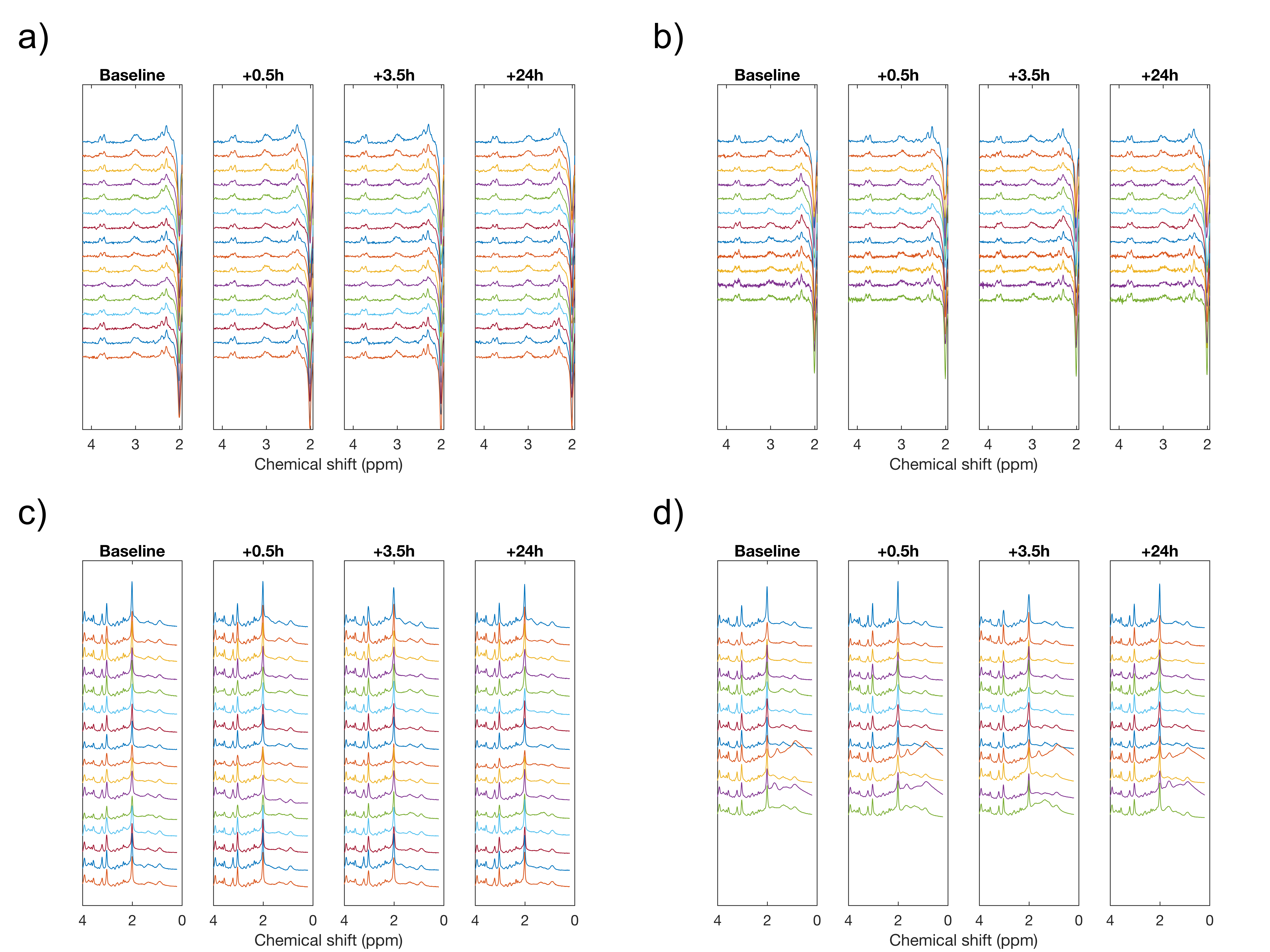


**Supplementary Figure 2.** Individual spectra of a) MEGA-PRESS scan and b) PRESS scan.

**
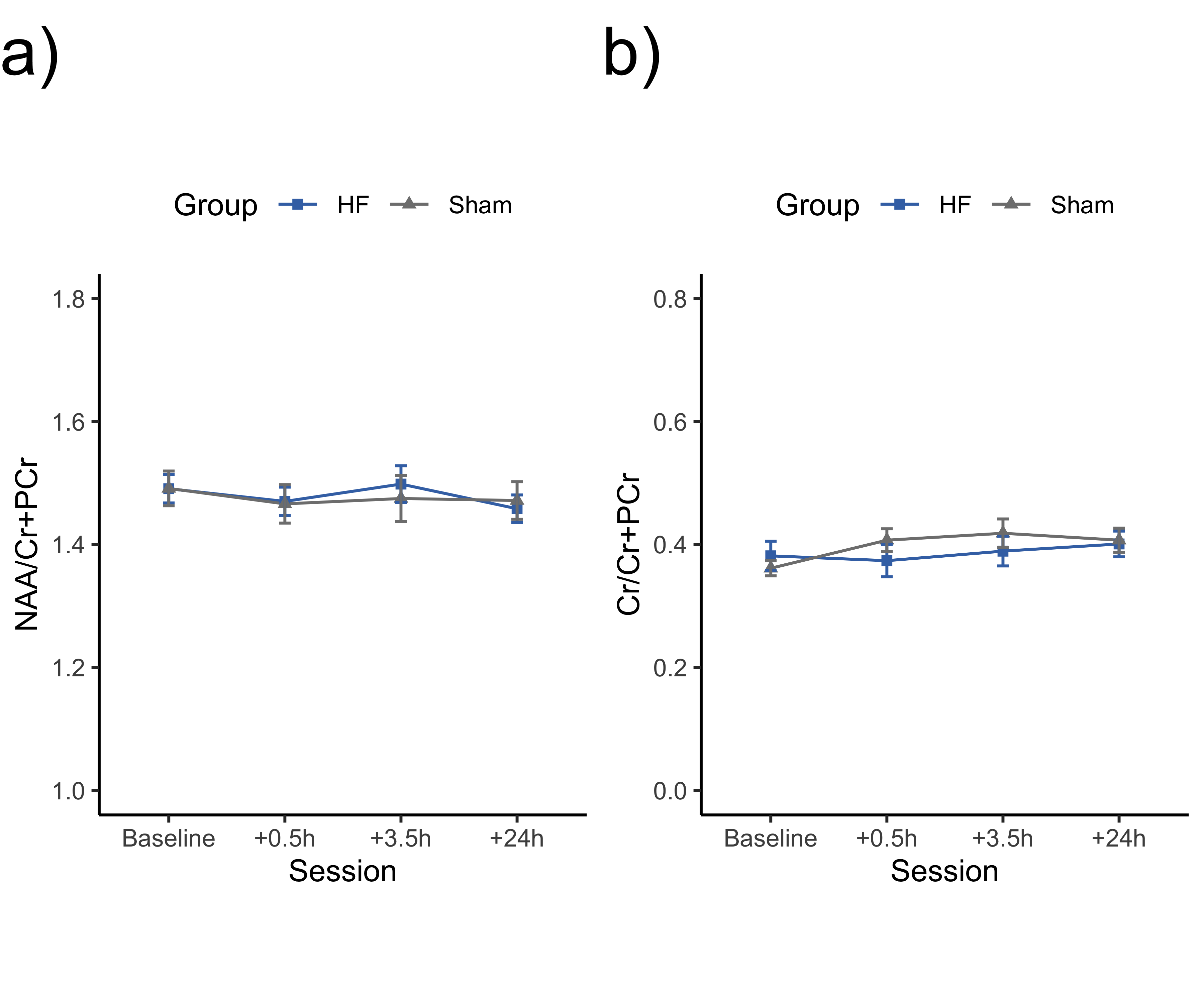
**

**Supplementary Figure 3.** Results of additional analyses of MRS data in Experiment 1. a) Mean (±s.e.m.) N-acetyl aspartate (NAA) concentrations in the HF rTMS (blue) and sham rTMS (gray) groups. We did not find any evidence that group or time significantly affected NAA concentrations. Results of a two-way mixed-model ANOVA on NAA concentrations with factors of time (baseline and 0.5 h, 3.5 h, and 24 h after rTMS intervention) and group (HF rTMS vs. sham rTMS) showed no significant main effect of group (*F*_1,26_ = 0.24, *p* = 0.628, *η*2 *p* = 0.005), time (*F*_3,78_ = 1.69, *p* = 0.175, *η*2 *p* = 0.026), or interaction between the two factors (*F*_3,78_ = 1.09, *p* = 0.357, *η*2 *p* = 0.117). b) Mean (±s.e.m.) Cr concentrations in the HF rTMS (blue) and sham rTMS (gray) groups. We did not find any evidence that group or time significantly affected Cr concentrations. Results of a two-way mixed-model ANOVA on Cr concentrations with factors of time (baseline and 0.5 h, 3.5 h, and 24 h after rTMS intervention) and group (HF rTMS vs. sham rTMS) showed no significant main effect of group (*F*_1,26_ = 0.01, *p* = 0.925, *η*2 *p* = 0.001), time (*F*_3,78_ = 1.63, *p* = 0.190, *η*2 *p* = 0.012), or interaction between the two factors (*F*_3,78_ = 0.56, *p* = 0.645, *η*2 *p* = 0.004).


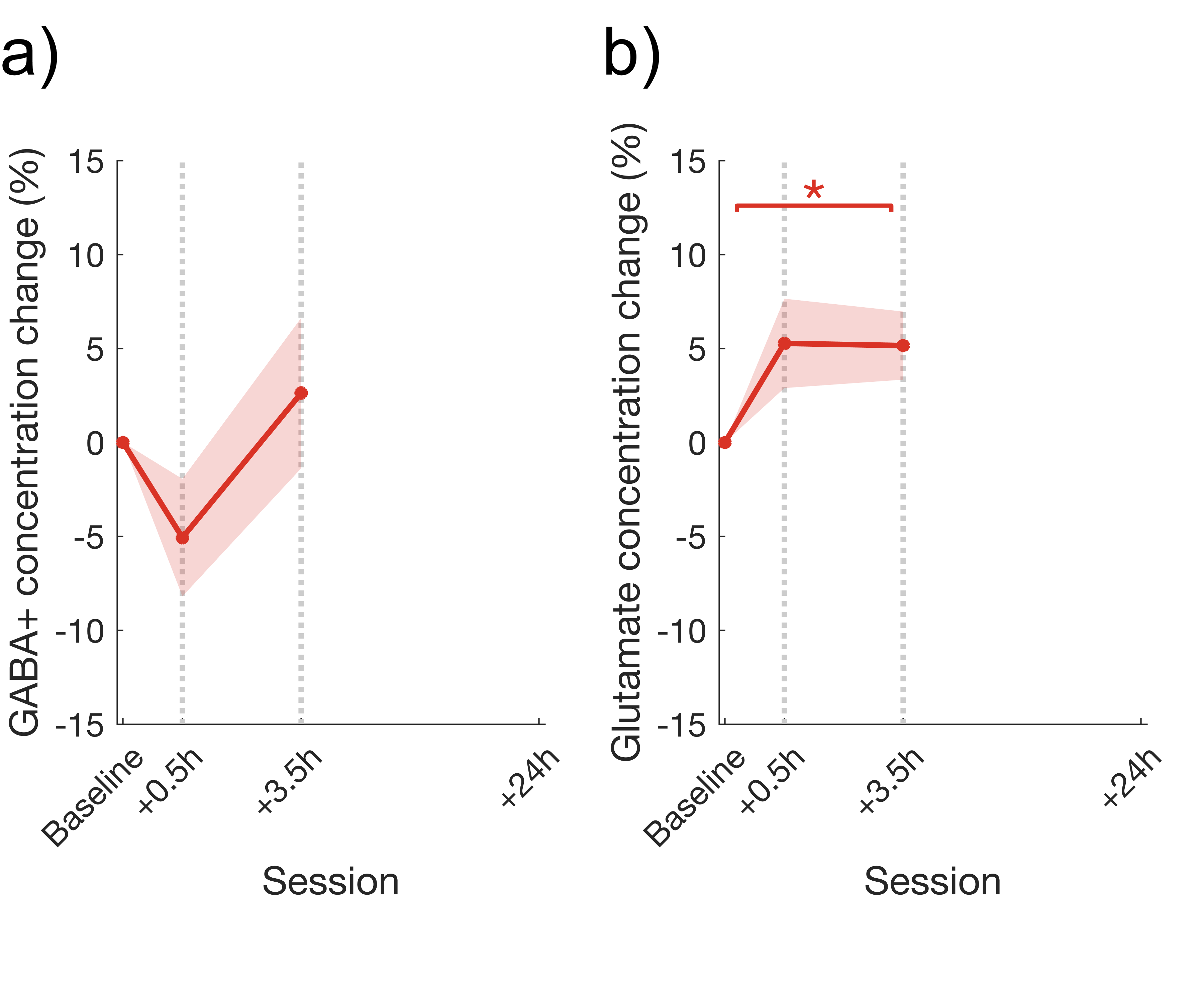


**Supplementary Figure 4.** Mean (± s.e.m.) changes in the concentrations of a) GABA+ and b) glutamate across the measured time course in the VPL group. a) We did not observe any significant difference in the GABA+ level. b) The mean ratio of glutamate was higher at 0.5 h (*p*=0.049, uncorrected) and 3.5 h (*p*=0.048, FDR corrected) when compared to baseline. Shaded areas indicate s.e.m. An asterisk indicates a significance level of **p* < 0.05 after FDR correction.

**Supplementary Table 1.** MRS quality.

|  | Time | HF  (n=16) | sham  (n=12) |  |
| --- | --- | --- | --- | --- |
| **MEGA-PRESS** | | | | |
| GABA+ %SD | Baseline | 6.31  (1.40) | 6.17  (1.12) | main effect of group:  *F*_1,26_=1.28, *p*= 0.269, *η*2 *p*=0.04 |
|  | 0.5 h | 6.44  (0.89) | 5.83 (0.84) | main effect of time:  *F*_3,78_=0.63, *p*=0.599, *η*2 *p*<0.01 |
|  | 3.5 h | 6.62  (0.81) | 6.08  (1.16) | group X time interaction:  *F*_3,78_=0.95, *p*=0.422, *η*2 *p*=0.01 |
|  | 24 h | 6.31  (0.87) | 6.08  (1.00) |  |
| FWHM (ppm) | Baseline | 0.06  (0.02) | 0.06  (0.01) | main effect of group:  *F*_1,26_= 2.53, *p*=0.124, *η*2 *p*=0.06 |
|  | 0.5 h | 0.06  (0.02) | 0.06  (0.02) | main effect of time:  *F*_3,78_= 0.41, *p*=0.747, *η*2 *p*<0.01 |
|  | 3.5 h | 0.07  (0.02) | 0.05  (0.02) | group X time interaction:  *F*_3,78_= 1.00, *p*=0.398, *η*2 *p*=0.01 |
|  | 24 h | 0.06  (0.02) | 0.06  (0.01) |  |
| SNR | Baseline | 25.8  (3.73) | 26.2  (2.95) | main effect of group:  *F*_1,26_=0.08, *p*=0.774, *η*2 *p*<0.01 |
|  | 0.5 h | 25.8  (3.62) | 26.5  (3.50) | main effect of time:  *F*_3,78_=0.30, *p*=0.828, *η*2 *p*<0.01 |
|  | 3.5 h | 25.7  (3.93) | 25.8  (2.41) | group X time interaction:  *F*_3,78_=0.18, *p*=0.910, *η*2 *p*<0.01 |
|  | 24 h | 26.2  (3.28) | 26.2  (2.90) |  |
| **PRESS** | | | | |
| Glutamate %SD | Baseline | 4.94  (0.80) | 5.58  (0.60) | main effect of group:  *F*_1,26_=6.13, *p*=0.020, *η*2 *p*=0.14 |
|  | 0.5 h | 5.06  (0.72) | 6.08  (1.00) | main effect of time:  *F*_3,78_=1.34, *p*=0.266, *η*2 *p*=0.02 |
|  | 3.5 h | 4.81  (0.62) | 5.75  (0.79) | group X time interaction:  *F*_3,78_=0.73, *p*=0.539, *η*2 *p*<0.01 |
|  | 24 h | 5.19  (0.55) | 5.75  (0.54) |  |
| Glx %SD | Baseline | 5.31 (0.70) | 6.33 (1.30) | main effect of group:  *F*_1,26_=6.79, *p*=0.015, *η*2 *p*=0.17 |
|  | 0.5 h | 5.56 (0.63) | 6.58 (1.62) | main effect of time:  *F*_3,78_=0.92, *p*=0.436, *η*2 *p*<0.01 |
|  | 3.5 h | 5.56 (0.63) | 6.42 (1.62) | group X time interaction:  *F*_3,78_=0.27, *p*=0.847, *η*2 *p*<0.01 |
|  | 24 h | 5.62 (0.62) | 6.42 (1.16) |  |
| NAA %SD | Baseline | 2 (0) | 2.08 (0.29) | main effect of group:  *F*_1,26_=1.75, *p*=0.197, *η*2 *p*=0.02 |
|  | 0.5 h | 1.94 (0.25) | 2.17 (0.39) | main effect of time:  *F*_2.4,63_=0.28, *p*=0.800, *η*2 *p*<0.01 |
|  | 3.5 h | 2.12 (0.34) | 2.08 (0.29) | group X time interaction:  *F*_2.4,63_=1.23, *p*=0.305, *η*2 *p*=0.03 |
|  | 24 h | 2.06 (0.25) | 2.08 (0.29) |  |
| FWHM (ppm) | Baseline | 0.05  (0.01) | 0.04  (0.01) | main effect of group:  *F*_1,26_=0.46, *p*=0.502, *η*2 *p*<0.01 |
|  | 0.5 h | 0.04  (0.01) | 0.04  (0.01) | main effect of time:  *F*_2.4,61.8_=0.71, *p*=0.518, *η*2 *p*=0.02 |
|  | 3.5 h | 0.04  (0.01) | 0.04  (0.01) | group X time interaction:  *F*_2.4,61.8_=0.49, *p*=0.648, *η*2 *p*=0.01 |
|  | 24 h | 0.04  (0.01) | 0.04  (0.01) |  |
| SNR | Baseline | 50.3  (5.56) | 50.3  (7.55) | main effect of group:  *F*_1,26_=0.21, *p*=0.652, *η*2 *p*<0.01 |
|  | 0.5 h | 50.2  (5.46) | 48.9  (6.39) | main effect of time:  *F*_2.3,60_=0.64, *p*=0.549, *η*2 *p*<0.01 |
|  | 3.5 h | 50.7  (6.61) | 48.8  (5.94) | group X time interaction:  *F*_2.3,60_=0.41, *p*=0.693, *η*2 *p*<0.01 |
|  | 24 h | 49.3 (6.71) | 48.8  (4.73) |  |

*Note*. Values are mean (± s.e.m.). %SD, estimated percentage standard deviations for CRLB; FWHM, full width at half maximum; SNR, signal-to-noise ratio.

**Supplementary Table 2.** MRSinMRS checklist.

| 1. Hardware |  |  |
| --- | --- | --- |
| a. Field strength [T] | 3 T | 3 T |
| b. Manufacturer | Siemens | Siemens |
| c. Model (software version if available) | Trio (VB17) | Trio (VB17) |
| d. RF coils: nuclei (transmit/receive), number of channels, type, body part | 32 channel ^1^H head coil | 32 channel ^1^H head coil |
| e. Additional hardware | N/A | N/A |
|  |  |  |
| 2. Acquisition |  |  |
| a. Pulse sequence | MEGA-PRESS | PRESS |
| b. Volume of interest (VOI) locations | visual cortex | visual cortex |
| c. Nominal VOI size [cm^3^, mm^3^] | 20 × 20 × 20 mm^3^ | 20 × 20 × 20 mm^3^ |
| d. Repetition time (TR), echo time (TE) [ms, s] | TR=1500 ms, TE= 68ms | TR=3000 ms, TE= 30ms |
| e. Total number of excitations or acquisitions per spectrum | 512 | 128 |
| f. Additional sequence parameters (bandwidth in Hz or dwell time in ms, number of spectral points, frequency offsets) | 2000 Hz, 2048 data points | 2500 Hz, 2048 data points |
| g. Water suppression method | CHESS | CHESS |
| h. Shimming method, reference peak, and thresholds for “acceptance of shim” chosen | Automated B0 field mapping followed by manual shimming of water | Automated B0 field mapping followed by manual shimming of water |
| i. Triggering or motion correction method (respiratory, peripheral, cardiac triggering, incl. device used and delays) | None | None |
|  |  |  |
| 3. Data analysis methods and outputs |  |  |
| a. Analysis software | LCModel 6.3 | LCModel 6.3 |
| b. Processing steps deviating from quoted reference or product | N/A | N/A |
| c. Output measure (eg absolute concentration, institutional units, ratio), processing steps deviating from quoted reference or product | Ratios to total NAA | Ratios to total creatine (for NAA and Cr level) |
| d. Quantification references and assumptions, fitting model assumptions | listed in Supplementary Figure 1 referencing to metabolite | listed in Supplementary Figure 1  referencing to metabolite |
|  |  |  |
| 4. Data quality |  |  |
| a. Reported variables (SNR, linewidth (with reference peaks)) | SNR (NAA): 26.0 ± 3.2  FWHM (NAA): 0.060 ± 0.016 ppm as reported by LCModel | SNR (NAA): 49.6 ± 6.0  FWHM (NAA): 0.043 ± 0.007 ppm as reported by LCModel |
| b. Data exclusion criteria  c. Quality measures of postprocessing model fitting (eg CRLB, goodness of fit, SD of residual) | SNR < 17;  FWHM > 0.1 ppm;  CRLB > 10% | SNR < 40;  FWHM > 0.1 ppm;  CRLB > 10% |
| d. Sample spectrum | Supplementary Figure 1 & 2 | Supplementary Figure 1 & 2 |

**Supplementary Table 3.** Stepwise forward model comparisons for fixed effects.

| Fixed Effect Term | df | AIC | BIC | χ^2^ | df | *p* |
| --- | --- | --- | --- | --- | --- | --- |
| Base model: Random intercepts for participant | 3 | 1164.0 | 1173.0 |  |  |  |
| + Group | 5 | 1164.5 | 1179.5 | 3.58 | 2 | 0.166 |
| + Time | 8 | 1165.4 | 1189.4 | 5.03 | 3 | 0.169 |
| + Group × Time | 13 | 1161.1 | 1200.6 | 13.82 | 5 | 0.016 * |

*Note*. ****p* < 0.001, ***p* < 0.01, **p* < 0.05.

**Supplementary Table 4.** Summary of Linear mixed-effect models of E/I ratio for the HF rTMS, sham rTMS, and VPL groups.

|  | **E/I ratio** | | |
| --- | --- | --- | --- |
| *Predictors* | *Estimates* | *CI (95%)* | *p* |
| Intercept | -0.00 | -5.77 – 5.77 | 1.000 |
| Time [0.5 h] | 2.17 | -5.45 – 9.79 | 0.576 |
| Time [3.5 h] | 11.49 | 3.87 – 19.11 | **0.003** |
| Time [24 h] | 4.13 | -3.49 – 11.75 | 0.288 |
| Group [VPL] | 0.00 | -8.82 – 8.82 | 1.000 |
| Group [Sham] | 0.00 | -8.82 – 8.82 | 1.000 |
| Time [0.5 h] * Group [VPL] | 9.80 | -1.84 – 21.44 | 0.099 |
| Time [3.5 h] * Group [VPL] | -7.76 | -19.40 – 3.88 | 0.191 |
| Time [0.5 h] * Group [Sham] | -1.09 | -12.73 – 10.55 | 0.855 |
| Time [3.5 h] * Group [Sham] | -14.33 | -25.97 – -2.69 | **0.016** |
| Time [24 h] * Group [Sham] | -1.24 | -12.88 – 10.40 | 0.834 |
| **Random Effects** | | | |
| σ^2^ | 120.93 | | |
| τ_00_ _ID_ | 17.81 | | |
| ICC | 0.13 | | |
| N _ID_ | 40 | | |
| Observations | 148 | | |
| Marginal R^2^ / Conditional R^2^ | 0.127 / 0.239 | | |

**Supplementary Table 5.** Summary of Linear mixed-effect models of E/I ratio for the HF rTMS and sham rTMS groups.

|  | **E/I ratio** | | |
| --- | --- | --- | --- |
| *Predictors* | *Estimates* | *CI (95%)* | *p* |
| (Intercept) | 0.00 | -5.99 – 5.99 | 1.000 |
| Time2 | 2.17 | -5.59 – 9.94 | 0.583 |
| Time3 | 11.49 | 3.72 – 19.26 | **0.004** |
| Time4 | 4.13 | -3.64 – 11.90 | 0.298 |
| GroupSham | -0.00 | -9.15 – 9.15 | 1.000 |
| Time2:GroupSham | -1.09 | -12.95 – 10.78 | 0.858 |
| Time3:GroupSham | -14.33 | -26.20 – -2.47 | **0.018** |
| Time4:GroupSham | -1.24 | -13.11 – 10.62 | 0.837 |
| **Random Effects** | | | |
| σ^2^ | 125.65 | | |
| τ_00_ _ID_ | 23.82 | | |
| ICC | 0.16 | | |
| N _ID_ | 28 | | |
| Observations | 112 | | |
| Marginal R^2^ / Conditional R^2^ | 0.102 / 0.245 | | |

**Supplementary Table 6.** Summary of Linear mixed-effect models of GABA+ for the HF rTMS, sham rTMS, and VPL groups.

|  | **GABA+** | | |
| --- | --- | --- | --- |
| *Predictors* | *Estimates* | *CI (95%)* | *p* |
| Intercept | -0.00 | -6.05 – 6.05 | 1.000 |
| Time [0.5 h] | -0.18 | -7.58 – 7.22 | 0.962 |
| Time [3.5 h] | -7.47 | -14.86 – -0.07 | **0.048** |
| Time [24 h] | -2.90 | -10.30 – 4.50 | 0.442 |
| Group [VPL] | 0.00 | -9.24 – 9.24 | 1.000 |
| Group [Sham] | 0.00 | -9.24 – 9.24 | 1.000 |
| Time [0.5 h] * Group [VPL] | -4.89 | -16.19 – 6.41 | 0.396 |
| Time [3.5 h] * Group [VPL] | 10.09 | -1.21 – 21.39 | 0.080 |
| Time [0.5 h] * Group [Sham] | 1.46 | -9.84 – 12.76 | 0.800 |
| Time [3.5 h] * Group [Sham] | 13.75 | 2.45 – 25.05 | **0.017** |
| Time [24 h] * Group [Sham] | 3.14 | -8.16 – 14.44 | 0.585 |
| **Random Effects** | | | |
| σ^2^ | 113.95 | | |
| τ_00_ _ID_ | 38.50 | | |
| ICC | 0.25 | | |
| N _ID_ | 40 | | |
| Observations | 148 | | |
| Marginal R^2^ / Conditional R^2^ | 0.076 / 0.309 | | |

**Supplementary Table 7.** Summary of Linear mixed-effect models of GABA+ for the HF rTMS and sham rTMS groups.

|  | **GABA+** | | |
| --- | --- | --- | --- |
| *Predictors* | *Estimates* | *CI (95%)* | *p* |
| (Intercept) | 0.00 | -6.37 – 6.37 | 1.000 |
| Time2 | -0.18 | -7.77 – 7.41 | 0.963 |
| Time3 | -7.47 | -15.05 – 0.12 | 0.054 |
| Time4 | -2.90 | -10.49 – 4.69 | 0.454 |
| GroupSham | -0.00 | -9.73 – 9.73 | 1.000 |
| Time2:GroupSham | 1.46 | -10.13 – 13.05 | 0.805 |
| Time3:GroupSham | 13.75 | 2.16 – 25.34 | **0.020** |
| Time4:GroupSham | 3.14 | -8.44 – 14.73 | 0.595 |
| **Random Effects** | | | |
| σ^2^ | 119.87 | | |
| τ_00_ _ID_ | 49.22 | | |
| ICC | 0.29 | | |
| N _ID_ | 28 | | |
| Observations | 112 | | |
| Marginal R^2^ / Conditional R^2^ | 0.073 / 0.343 | | |

**Supplementary Table 8.** Summary of Linear mixed-effect models of glutamate for the HF rTMS, sham rTMS, and VPL groups.

|  | **Glutamate** | | |
| --- | --- | --- | --- |
| *Predictors* | *Estimates* | *CI (95%)* | *p* |
| Intercept | -0.00 | -3.54 – 3.54 | 1.000 |
| Time [0.5 h] | -0.26 | -4.47 – 3.95 | 0.903 |
| Time [3.5 h] | 2.06 | -2.15 – 6.27 | 0.338 |
| Time [24 h] | 0.46 | -3.75 – 4.67 | 0.830 |
| Group [VPL] | 0.00 | -5.41 – 5.41 | 1.000 |
| Group [Sham] | 0.00 | -5.41 – 5.41 | 1.000 |
| Time [0.5 h] * Group [VPL] | 5.53 | -0.90 – 11.96 | 0.092 |
| Time [3.5 h] * Group [VPL] | 3.09 | -3.34 – 9.52 | 0.346 |
| Time [0.5 h] * Group [Sham] | 0.44 | -5.99 – 6.87 | 0.894 |
| Time [3.5 h] * Group [Sham] | -0.02 | -6.45 – 6.41 | 0.994 |
| Time [24 h] * Group [Sham] | 0.67 | -5.76 – 7.10 | 0.838 |
| **Random Effects** | | | |
| σ^2^ | 36.89 | | |
| τ_00_ _ID_ | 15.39 | | |
| ICC | 0.29 | | |
| N _ID_ | 40 | | |
| Observations | 148 | | |
| Marginal R^2^ / Conditional R^2^ | 0.063 / 0.339 | | |

**Supplementary Table 9.** Summary of Linear mixed-effect models of glutamate for the HF rTMS and sham rTMS groups.

|  | **Glutamate** | | |
| --- | --- | --- | --- |
| *Predictors* | *Estimates* | *CI (95%)* | *p* |
| (Intercept) | 0.00 | -3.73 – 3.73 | 1.000 |
| Time2 | -0.26 | -4.64 – 4.11 | 0.907 |
| Time3 | 2.06 | -2.32 – 6.43 | 0.357 |
| Time4 | 0.46 | -3.91 – 4.84 | 0.836 |
| GroupSham | -0.00 | -5.69 – 5.69 | 1.000 |
| Time2:GroupSham | 0.44 | -6.24 – 7.12 | 0.898 |
| Time3:GroupSham | -0.02 | -6.71 – 6.66 | 0.995 |
| Time4:GroupSham | 0.67 | -6.01 – 7.35 | 0.844 |
| **Random Effects** | | | |
| σ^2^ | 39.86 | | |
| τ_00_ _ID_ | 17.99 | | |
| ICC | 0.31 | | |
| N _ID_ | 28 | | |
| Observations | 112 | | |
| Marginal R^2^ / Conditional R^2^ | 0.013 / 0.320 | | |
